# *trans*-Bis(benzyl­diphenyl­phosphane-κ*P*)dichloridoplatinum(II)

**DOI:** 10.1107/S160053681204696X

**Published:** 2012-11-24

**Authors:** Alfred Muller

**Affiliations:** a Research Centre for Synthesis and Catalysis, Department of Chemistry, University of Johannesburg (APK Campus), PO Box 524, Auckland Park, Johannesburg, 2006, South Africa

## Abstract

In the mononuclear title compound, *trans*-[PtCl_2_(C_19_H_17_P)_2_], the slightly distorted square-planar coordination sphere of the Pt^II^ atom is occupied by two benzyl­diphenyl­phosphane ligands and two chloride atoms in a mutually *trans* geometry. The effective cone angles for the two phosphane ligands are 160 and 169°. C—H⋯Cl inter­actions generate infinite long chains along [01-1]. Additional C—H⋯π and π–π stacking interactions [centroid–centroid distance = 4.2499 (15) Å and ring slippage = 2.386 Å] are observed.

## Related literature

For reviews of related compounds, see: Spessard & Miessler (1996 ▶); Muller & Meijboom (2010 ▶). For background to cone angles, see: Tolman (1977 ▶); Otto (2001 ▶). For the *cis* isomer of the title compound, see: Davis & Meijboom (2011 ▶).
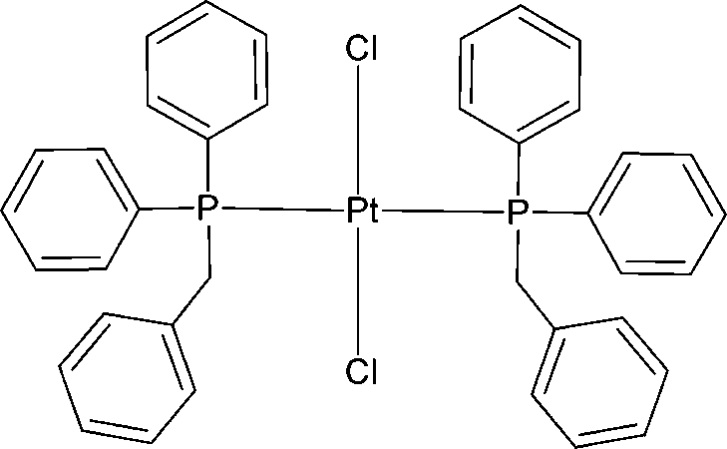


## Experimental

### 

#### Crystal data

[PtCl_2_(C_19_H_17_P)_2_]
*M* *_r_* = 818.58
Triclinic, 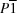

*a* = 9.5585 (12) Å
*b* = 13.4135 (17) Å
*c* = 14.7553 (18) Å
α = 66.307 (2)°
β = 73.147 (3)°
γ = 88.034 (3)°
*V* = 1650.7 (4) Å^3^
*Z* = 2
Mo *K*α radiation
μ = 4.54 mm^−1^
*T* = 100 K
0.24 × 0.1 × 0.08 mm

#### Data collection

Bruker APEX DUO 4K CCD diffractometer
Absorption correction: multi-scan (*SADABS*; Bruker, 2008 ▶) *T*_min_ = 0.409, *T*_max_ = 0.713
33198 measured reflections
8253 independent reflections
7779 reflections with *I* > 2σ(*I*)
*R*_int_ = 0.031

#### Refinement

*R*[*F* ^2^ > 2σ(*F* ^2^)] = 0.018
*wR*(*F* ^2^) = 0.039
*S* = 1.03
8253 reflections
388 parameters
H-atom parameters constrained
Δρ_max_ = 0.65 e Å^−3^
Δρ_min_ = −0.54 e Å^−3^

### 

Data collection: *APEX2* (Bruker, 2011 ▶); cell refinement: *SAINT* (Bruker, 2008 ▶); data reduction: *SAINT* and *XPREP* (Bruker, 2008 ▶); program(s) used to solve structure: *SIR97* (Altomare *et al.*, 1999 ▶); program(s) used to refine structure: *SHELXL97* (Sheldrick, 2008 ▶); molecular graphics: *DIAMOND* (Brandenburg & Putz, 2005 ▶); software used to prepare material for publication: *publCIF* (Westrip, 2010 ▶) and *WinGX* (Farrugia, 2012) ▶.

## Supplementary Material

Crystal structure: contains datablock(s) global, I. DOI: 10.1107/S160053681204696X/ng5305sup1.cif

Structure factors: contains datablock(s) I. DOI: 10.1107/S160053681204696X/ng5305Isup2.hkl

Additional supplementary materials:  crystallographic information; 3D view; checkCIF report

## Figures and Tables

**Table 1:** Hydrogen-bond geometry (Å, °) *Cg*1, *Cg*2, *Cg*3 and *Cg*4 are the centroids of the C33—C38, C2—C7, C8—C13 and C27—C32 rings, repectively.

*D*—H⋯*A*	*D*—H	H⋯*A*	*D*⋯*A*	*D*—H⋯*A*
C7—H7⋯Cl2^i^	0.95	2.85	3.532 (2)	130
C26—H26⋯Cl2	0.95	2.72	3.555 (2)	147
C29—H29⋯Cl1^ii^	0.95	2.93	3.731 (2)	143
C16—H16⋯*Cg*1^ii^	0.95	2.73	3.443 (3)	132
C23—H23⋯*Cg*2^iii^	0.95	2.63	3.536 (2)	159
C29—H29⋯*Cg*3^ii^	0.95	2.99	3.525 (2)	117
C36—H36⋯*Cg*4^iv^	0.95	2.77	3.708 (3)	169

Symmetry codes: (i) 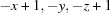; (ii) 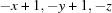; (iii) 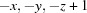; (iv) 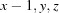.

**Fig. 1.**
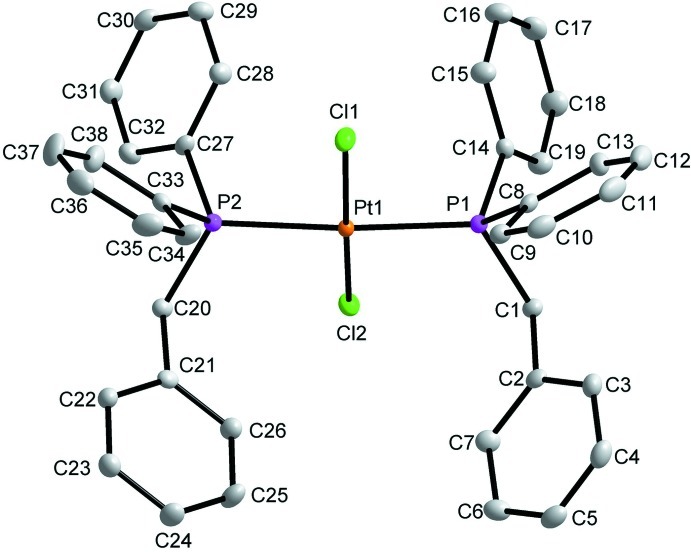
A view of the title complex, showing the atom-numbering scheme and 50% probability displacement ellipsoids. Hydrogen atoms have been omitted for clarity.

**Fig. 2.**
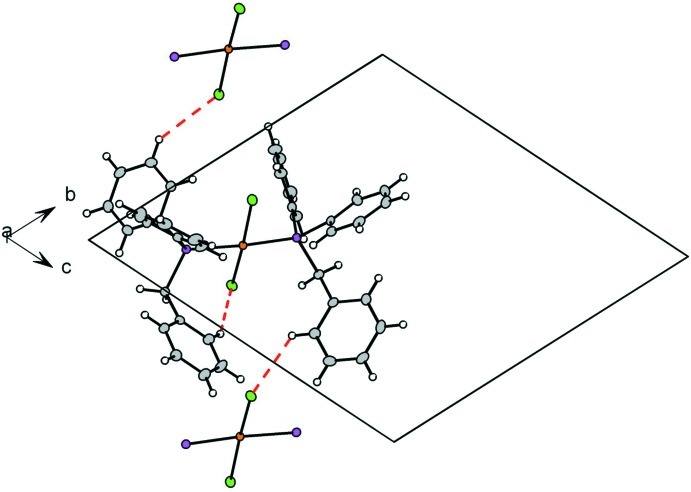
Packing diagram showing only the C—H···Cl interactions (indicated by red dashed lines).

**Fig. 3.**
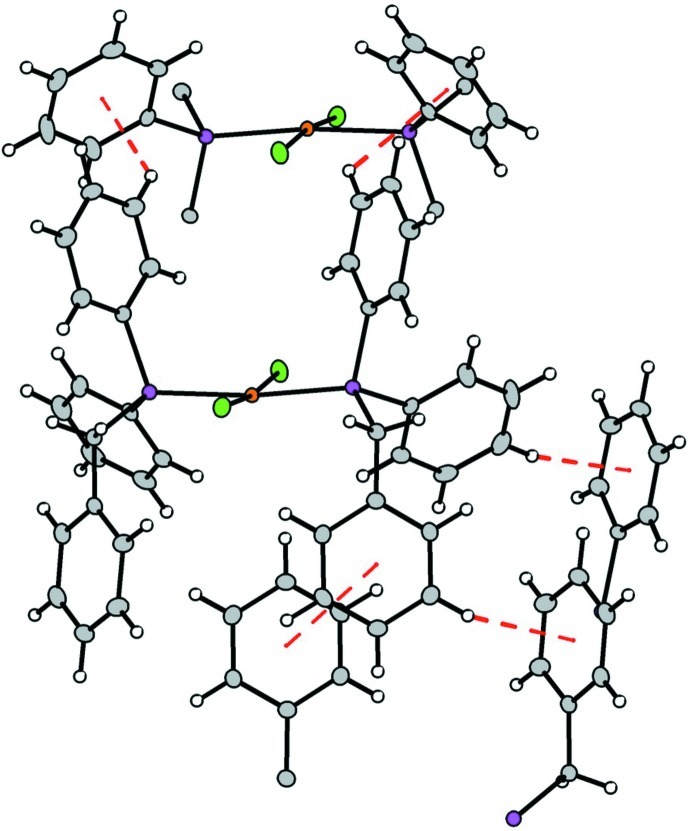
Packing diagram showing only the C—H···π interactions as well as π···π stacking (both indicated by red dashed lines).

## supplementary crystallographic information

### Comment

Transition metal complexes containing phosphine, arsine and stibine ligands are widely being investigated in various fields of organometallic chemistry (Spessard & Miessler, 1996). As part of a systematic investigation (Muller & Meijboom, 2010) involving complexes with the general formula *trans/cis*-[*MX*_2_(*L*)_2_] (*M* = Pt or Pd; *X* = halogen, Me, Ph; *L* = Group 15 donor ligand), crystals of the title compound were obtained by reaction of Zeize's salt, K[Pt(η^2^-ethylene)Cl_3_], with benzyldiphenylphosphane.

Molecules of the title compound (Fig. 1) crystallizes in the *P*1 (*Z* = 2) space group with the Pt atom lying on general positions in the unit cell. Each pair of equivalent ligands is in a mutually *trans* orientation with only slight distortion observed in the Pt square-planar coordination sphere (An averaged plane formed by the ligand donor atoms reveals that the Pt is displaced 0.1314 (3) Å from it; r.m.s. of fitted atoms = 0.0041 Å). The Pt—P1/P2 distances differ marginally (2.3096 (5) and 2.3155 (5) Å respectively), while the Pt—Cl distances deviates even less (2.3102 (5) and 2.3128 (5) Å respectively). The P—Pt—P and Cl—Pt—Cl angles show similar distortions from linearity (173.556 (18) and 173.220 (18)° respectively). The orientation of the phosphanes is such that they appear eclipsed when viewed along the P—M–P axis with one of the cyclohexcyl substituents from each phosphane almost perpendicular to the metal square-planar coordination plane (C—P—Pt—Cl dihedral angles vary from 83.74 (7) to 89.59 (7) °). To investigate the steric demand of the phosphane ligands their cone angles were calculated using an adaptation of the Tolman cone angle model (Tolman, 1977) where the geometry form the crystal structure determination is used and the metal phosphorus distance adjusted to 2.28 Å (Otto, 2001). Values obtained with this method vary from 160 to 169°. Surprisingly the *cis* isomer (Davis & Meijboom, 2011) of the title compound, where more crowding of the bulky ligands are expected, have slightly larger cone angle values (calculation results vary from 172 to 177°) which attest to the flexibility of this particular ligands' substituents. Comparing the title compound to structures from literature where the benzyldiphenylphosphane is coordinated to other transition metals, the cone angle values are comparable to the average cone angle value calculated. Data extracted from the Cambridge Structural Database shows an average cone angle of 165° for the phosphane from 27 hits, containing 43 useable observations, with a standard deviation of ±12° and a spread from 140° to 184°.

In the crystal structure several C—H···Cl interactions are observed (see table 1 and Fig. 2) linking molecules in infinite long chains along the [01–1] direction. Additional C—H···π interactions as well as π···π stacking are observed (centroid to centroid distance = 4.2499 (15) Å, ring slippage = 2.386 Å). These are summarized in Table 1 and Fig. 3.

### Experimental

Potassium trichloro(ethylene)platinate(II) (10 mg, 0.0271 mmol) and benzyldiphenylphosphane (7.5 mg, 0.0271 mmol) were dissolved seperately in acetone (10 ml) and the latter added drop-wise to the other with stirring at room temperature (10 min). Slow evaporation of the solvent gave colourless crystals of the title compound suitable for a single-crystal X-ray diffraction study. Analytical data: ^31^P {H} NMR (CDCl_3_, 161.99 MHz): d = 13.58 (t, ^1^*J*(^31^P-^195^Pt) = 2308 Hz).

### Refinement

The aromatic and methylene H atoms were placed in geometrically idealized positions (C—H = 0.95–0.99) and allowed to ride on their parent atoms, with *U*_iso_(H) = 1.2*U*_eq_(C). The highest residual electron density (< 1 Å^3^) are within 1 Å from Pt and represent no physical meaning.

### Crystal data

**Table:**

[PtCl_2_(C_19_H_17_P)_2_]	*Z* = 2
*M**_r_* = 818.58	*F*(000) = 808
Triclinic, *P*1	*D*_x_ = 1.647 Mg m^−^^3^*D*_m_ = 1.647 Mg m^−^^3^*D*_m_ measured by not measured
Hall symbol: -P 1	Mo *K*α radiation, λ = 0.71073 Å
*a* = 9.5585 (12) Å	Cell parameters from 9225 reflections
*b* = 13.4135 (17) Å	θ = 2.6–28.4°
*c* = 14.7553 (18) Å	µ = 4.54 mm^−^^1^
α = 66.307 (2)°	*T* = 100 K
β = 73.147 (3)°	Needle, colourless
γ = 88.034 (3)°	0.24 × 0.1 × 0.08 mm
*V* = 1650.7 (4) Å^3^	

### Data collection

**Table:**

Bruker APEX DUO 4K CCD diffractometer	8253 independent reflections
Radiation source: sealed tube	7779 reflections with *I* > 2σ(*I*)
Graphite monochromator	*R*_int_ = 0.031
Detector resolution: 8.4 pixels mm^-1^	θ_max_ = 28.4°, θ_min_ = 1.6°
φ and ω scans	*h* = −12→12
Absorption correction: multi-scan (*SADABS*; Bruker, 2008)	*k* = −17→17
*T*_min_ = 0.409, *T*_max_ = 0.713	*l* = −19→19
33198 measured reflections	

### Refinement

**Table:**

Refinement on *F*^2^	Primary atom site location: structure-invariant direct methods
Least-squares matrix: full	Secondary atom site location: difference Fourier map
*R*[*F*^2^ > 2σ(*F*^2^)] = 0.018	Hydrogen site location: inferred from neighbouring sites
*wR*(*F*^2^) = 0.039	H-atom parameters constrained
*S* = 1.03	*w* = 1/[σ^2^(*F*_o_^2^) + (0.0157*P*)^2^ + 0.6462*P*] where *P* = (*F*_o_^2^ + 2*F*_c_^2^)/3
8253 reflections	(Δ/σ)_max_ = 0.002
388 parameters	Δρ_max_ = 0.65 e Å^−^^3^
0 restraints	Δρ_min_ = −0.54 e Å^−^^3^

### Special details

**Table:**

Geometry. All e.s.d.'s (except the e.s.d. in the dihedral angle between two l.s. planes) are estimated using the full covariance matrix. The cell e.s.d.'s are taken into account individually in the estimation of e.s.d.'s in distances, angles and torsion angles; correlations between e.s.d.'s in cell parameters are only used when they are defined by crystal symmetry. An approximate (isotropic) treatment of cell e.s.d.'s is used for estimating e.s.d.'s involving l.s. planes.
Refinement. Refinement of *F*^2^ against ALL reflections. The weighted *R*-factor *wR* and goodness of fit *S* are based on *F*^2^, conventional *R*-factors *R* are based on *F*, with *F* set to zero for negative *F*^2^. The threshold expression of *F*^2^ > σ(*F*^2^) is used only for calculating *R*-factors(gt) *etc*. and is not relevant to the choice of reflections for refinement. *R*-factors based on *F*^2^ are statistically about twice as large as those based on *F*, and *R*- factors based on ALL data will be even larger.

### Fractional atomic coordinates and isotropic or equivalent isotropic displacement parameters (Å^2^)

**Table:**

	*x*	*y*	*z*	*U*_iso_*/*U*_eq_	
Pt1	0.435237 (8)	0.254343 (6)	0.260155 (5)	0.00938 (2)	
Cl1	0.32010 (6)	0.39062 (4)	0.16068 (4)	0.01605 (10)	
Cl2	0.57602 (5)	0.12862 (4)	0.34467 (4)	0.01508 (9)	
P1	0.49965 (6)	0.36874 (4)	0.32689 (4)	0.01070 (10)	
P2	0.39698 (6)	0.14486 (4)	0.18021 (4)	0.01132 (10)	
C1	0.5284 (2)	0.30979 (16)	0.45596 (14)	0.0133 (4)	
H1A	0.5961	0.252	0.4572	0.016*	
H1B	0.579	0.3679	0.4636	0.016*	
C2	0.3947 (2)	0.26114 (16)	0.54973 (14)	0.0136 (4)	
C3	0.3107 (2)	0.32666 (17)	0.59474 (16)	0.0173 (4)	
H3	0.3307	0.4039	0.5611	0.021*	
C4	0.1986 (2)	0.27982 (18)	0.68797 (16)	0.0196 (4)	
H4	0.1428	0.325	0.7178	0.023*	
C5	0.1682 (2)	0.16662 (18)	0.73760 (16)	0.0194 (4)	
H5	0.093	0.1344	0.8021	0.023*	
C6	0.2477 (2)	0.10114 (17)	0.69279 (16)	0.0191 (4)	
H6	0.2257	0.024	0.7261	0.023*	
C7	0.3599 (2)	0.14780 (16)	0.59903 (15)	0.0163 (4)	
H7	0.413	0.1023	0.5684	0.02*	
C8	0.3766 (2)	0.47250 (15)	0.33790 (14)	0.0129 (4)	
C9	0.2264 (2)	0.44601 (16)	0.36676 (15)	0.0155 (4)	
H9	0.1906	0.3779	0.3725	0.019*	
C10	0.1284 (3)	0.51857 (18)	0.38729 (16)	0.0206 (5)	
H10	0.0257	0.5004	0.4063	0.025*	
C11	0.1805 (3)	0.61791 (18)	0.38000 (16)	0.0232 (5)	
H11	0.1135	0.667	0.3955	0.028*	
C12	0.3307 (3)	0.64529 (17)	0.34999 (17)	0.0233 (5)	
H12	0.3664	0.7135	0.3443	0.028*	
C13	0.4290 (3)	0.57345 (16)	0.32830 (16)	0.0178 (4)	
H13	0.5317	0.5928	0.307	0.021*	
C14	0.6772 (2)	0.44094 (15)	0.24140 (15)	0.0130 (4)	
C15	0.6881 (2)	0.50991 (17)	0.13829 (16)	0.0186 (4)	
H15	0.6028	0.5205	0.116	0.022*	
C16	0.8220 (3)	0.56263 (18)	0.06883 (17)	0.0220 (5)	
H16	0.8277	0.6108	−0.0004	0.026*	
C17	0.9486 (2)	0.54559 (18)	0.09962 (17)	0.0210 (5)	
H17	1.0408	0.5812	0.0515	0.025*	
C18	0.9389 (2)	0.47609 (18)	0.20123 (17)	0.0212 (5)	
H18	1.025	0.4641	0.2225	0.025*	
C19	0.8044 (2)	0.42395 (17)	0.27196 (16)	0.0182 (4)	
H19	0.799	0.3766	0.3413	0.022*	
C27	0.5508 (2)	0.17383 (16)	0.06288 (14)	0.0132 (4)	
C32	0.6258 (2)	0.09293 (17)	0.03745 (16)	0.0179 (4)	
H32	0.5979	0.018	0.0827	0.021*	
C31	0.7414 (2)	0.12179 (17)	−0.05392 (16)	0.0192 (4)	
H31	0.7925	0.0665	−0.0707	0.023*	
C30	0.7826 (2)	0.23148 (17)	−0.12105 (15)	0.0180 (4)	
H30	0.8613	0.251	−0.1836	0.022*	
C29	0.7083 (2)	0.31196 (17)	−0.09620 (15)	0.0182 (4)	
H29	0.7354	0.3867	−0.1422	0.022*	
C28	0.5946 (2)	0.28397 (16)	−0.00462 (15)	0.0169 (4)	
H28	0.5458	0.3398	0.0126	0.02*	
C33	0.2343 (2)	0.16194 (15)	0.13646 (15)	0.0138 (4)	
C38	0.2353 (3)	0.15109 (17)	0.04602 (17)	0.0205 (5)	
H38	0.3239	0.1398	0.0028	0.025*	
C37	0.1054 (3)	0.1569 (2)	0.01952 (19)	0.0282 (5)	
H37	0.1057	0.1486	−0.0415	0.034*	
C36	−0.0239 (3)	0.17476 (18)	0.08137 (17)	0.0239 (5)	
H36	−0.1117	0.1786	0.0627	0.029*	
C35	−0.0251 (2)	0.18690 (18)	0.17016 (17)	0.0225 (5)	
H35	−0.1135	0.1996	0.2125	0.027*	
C34	0.1039 (2)	0.18053 (18)	0.19755 (16)	0.0198 (4)	
H34	0.1027	0.189	0.2587	0.024*	
C20	0.3854 (2)	−0.00427 (15)	0.25407 (15)	0.0139 (4)	
H20A	0.3845	−0.0401	0.2072	0.017*	
H20B	0.4752	−0.0223	0.2758	0.017*	
C21	0.2533 (2)	−0.05230 (15)	0.34940 (15)	0.0133 (4)	
C26	0.2527 (2)	−0.05270 (17)	0.44434 (15)	0.0173 (4)	
H26	0.3344	−0.0185	0.4488	0.021*	
C25	0.1336 (3)	−0.10261 (19)	0.53223 (16)	0.0231 (5)	
H25	0.1345	−0.1027	0.5965	0.028*	
C24	0.0128 (2)	−0.15251 (18)	0.52703 (16)	0.0203 (4)	
H24	−0.0683	−0.1871	0.5875	0.024*	
C23	0.0117 (2)	−0.15134 (17)	0.43293 (16)	0.0184 (4)	
H23	−0.0705	−0.1851	0.4287	0.022*	
C22	0.1304 (2)	−0.10102 (16)	0.34484 (15)	0.0167 (4)	
H22	0.1279	−0.0997	0.2805	0.02*	

### Atomic displacement parameters (Å^2^)

**Table:**

	*U*^11^	*U*^22^	*U*^33^	*U*^12^	*U*^13^	*U*^23^
Pt1	0.00944 (4)	0.00964 (4)	0.00983 (4)	0.00115 (2)	−0.00353 (3)	−0.00432 (3)
Cl1	0.0197 (3)	0.0158 (2)	0.0176 (2)	0.00698 (19)	−0.0111 (2)	−0.00844 (18)
Cl2	0.0158 (2)	0.0131 (2)	0.0189 (2)	0.00403 (18)	−0.00962 (19)	−0.00633 (18)
P1	0.0105 (2)	0.0112 (2)	0.0106 (2)	0.00044 (18)	−0.00338 (19)	−0.00455 (18)
P2	0.0119 (3)	0.0115 (2)	0.0102 (2)	−0.00023 (19)	−0.00265 (19)	−0.00438 (18)
C1	0.0133 (10)	0.0147 (9)	0.0117 (9)	0.0004 (8)	−0.0048 (8)	−0.0046 (7)
C2	0.0139 (10)	0.0171 (9)	0.0105 (8)	0.0010 (8)	−0.0062 (8)	−0.0046 (7)
C3	0.0201 (11)	0.0181 (10)	0.0171 (10)	0.0013 (8)	−0.0070 (9)	−0.0095 (8)
C4	0.0183 (11)	0.0269 (11)	0.0200 (10)	0.0054 (9)	−0.0067 (9)	−0.0155 (9)
C5	0.0149 (11)	0.0285 (11)	0.0134 (9)	−0.0006 (9)	−0.0037 (8)	−0.0074 (8)
C6	0.0171 (11)	0.0179 (10)	0.0177 (10)	0.0005 (8)	−0.0052 (9)	−0.0027 (8)
C7	0.0122 (10)	0.0180 (10)	0.0171 (9)	0.0033 (8)	−0.0037 (8)	−0.0063 (8)
C8	0.0162 (10)	0.0139 (9)	0.0099 (8)	0.0034 (8)	−0.0046 (8)	−0.0060 (7)
C9	0.0171 (11)	0.0163 (9)	0.0119 (9)	0.0006 (8)	−0.0028 (8)	−0.0055 (7)
C10	0.0186 (11)	0.0271 (11)	0.0138 (9)	0.0069 (9)	−0.0030 (8)	−0.0079 (8)
C11	0.0315 (14)	0.0231 (11)	0.0164 (10)	0.0127 (10)	−0.0058 (9)	−0.0112 (9)
C12	0.0383 (15)	0.0157 (10)	0.0199 (10)	0.0062 (9)	−0.0118 (10)	−0.0094 (8)
C13	0.0223 (12)	0.0155 (10)	0.0183 (10)	0.0008 (8)	−0.0095 (9)	−0.0073 (8)
C14	0.0128 (10)	0.0128 (9)	0.0139 (9)	−0.0001 (7)	−0.0029 (8)	−0.0066 (7)
C15	0.0170 (11)	0.0198 (10)	0.0169 (10)	−0.0003 (8)	−0.0065 (8)	−0.0043 (8)
C16	0.0220 (12)	0.0215 (11)	0.0168 (10)	−0.0037 (9)	−0.0052 (9)	−0.0023 (8)
C17	0.0161 (11)	0.0218 (11)	0.0203 (10)	−0.0048 (9)	0.0008 (9)	−0.0078 (9)
C18	0.0142 (11)	0.0262 (11)	0.0229 (11)	0.0000 (9)	−0.0067 (9)	−0.0088 (9)
C19	0.0173 (11)	0.0193 (10)	0.0170 (10)	0.0009 (8)	−0.0068 (8)	−0.0053 (8)
C27	0.0108 (10)	0.0177 (9)	0.0109 (8)	−0.0008 (8)	−0.0016 (7)	−0.0068 (7)
C32	0.0206 (11)	0.0139 (9)	0.0174 (10)	−0.0004 (8)	−0.0040 (9)	−0.0056 (8)
C31	0.0199 (12)	0.0189 (10)	0.0197 (10)	0.0030 (9)	−0.0036 (9)	−0.0107 (8)
C30	0.0143 (11)	0.0249 (11)	0.0128 (9)	0.0003 (8)	−0.0013 (8)	−0.0073 (8)
C29	0.0181 (11)	0.0168 (10)	0.0148 (9)	−0.0005 (8)	−0.0027 (8)	−0.0031 (8)
C28	0.0185 (11)	0.0153 (9)	0.0160 (9)	0.0022 (8)	−0.0033 (8)	−0.0070 (8)
C33	0.0152 (10)	0.0108 (9)	0.0147 (9)	−0.0023 (7)	−0.0058 (8)	−0.0036 (7)
C38	0.0261 (12)	0.0218 (11)	0.0203 (10)	0.0071 (9)	−0.0104 (9)	−0.0132 (9)
C37	0.0375 (15)	0.0330 (13)	0.0278 (12)	0.0054 (11)	−0.0217 (11)	−0.0180 (10)
C36	0.0221 (12)	0.0231 (11)	0.0263 (11)	−0.0044 (9)	−0.0147 (10)	−0.0043 (9)
C35	0.0130 (11)	0.0274 (11)	0.0197 (10)	−0.0026 (9)	−0.0027 (9)	−0.0034 (9)
C34	0.0168 (11)	0.0274 (11)	0.0118 (9)	−0.0046 (9)	−0.0032 (8)	−0.0049 (8)
C20	0.0153 (10)	0.0125 (9)	0.0130 (9)	0.0015 (8)	−0.0033 (8)	−0.0051 (7)
C21	0.0151 (10)	0.0098 (8)	0.0132 (9)	0.0025 (7)	−0.0034 (8)	−0.0037 (7)
C26	0.0171 (11)	0.0190 (10)	0.0158 (9)	−0.0020 (8)	−0.0059 (8)	−0.0063 (8)
C25	0.0237 (12)	0.0324 (12)	0.0131 (9)	−0.0044 (10)	−0.0041 (9)	−0.0096 (9)
C24	0.0164 (11)	0.0257 (11)	0.0159 (10)	−0.0043 (9)	−0.0002 (8)	−0.0084 (8)
C23	0.0175 (11)	0.0189 (10)	0.0191 (10)	−0.0017 (8)	−0.0053 (9)	−0.0079 (8)
C22	0.0206 (11)	0.0165 (10)	0.0135 (9)	−0.0019 (8)	−0.0055 (8)	−0.0059 (8)

### Geometric parameters (Å, º)

**Table:**

Pt1—P1	2.3096 (5)	C17—C18	1.388 (3)
Pt1—Cl2	2.3102 (5)	C17—H17	0.95
Pt1—Cl1	2.3128 (5)	C18—C19	1.388 (3)
Pt1—P2	2.3155 (5)	C18—H18	0.95
P1—C14	1.817 (2)	C19—H19	0.95
P1—C8	1.818 (2)	C27—C32	1.397 (3)
P1—C1	1.8456 (19)	C27—C28	1.403 (3)
P2—C33	1.824 (2)	C32—C31	1.391 (3)
P2—C27	1.825 (2)	C32—H32	0.95
P2—C20	1.8424 (19)	C31—C30	1.394 (3)
C1—C2	1.510 (3)	C31—H31	0.95
C1—H1A	0.99	C30—C29	1.385 (3)
C1—H1B	0.99	C30—H30	0.95
C2—C7	1.399 (3)	C29—C28	1.385 (3)
C2—C3	1.403 (3)	C29—H29	0.95
C3—C4	1.390 (3)	C28—H28	0.95
C3—H3	0.95	C33—C34	1.391 (3)
C4—C5	1.393 (3)	C33—C38	1.396 (3)
C4—H4	0.95	C38—C37	1.397 (3)
C5—C6	1.384 (3)	C38—H38	0.95
C5—H5	0.95	C37—C36	1.385 (4)
C6—C7	1.395 (3)	C37—H37	0.95
C6—H6	0.95	C36—C35	1.381 (3)
C7—H7	0.95	C36—H36	0.95
C8—C9	1.388 (3)	C35—C34	1.394 (3)
C8—C13	1.398 (3)	C35—H35	0.95
C9—C10	1.388 (3)	C34—H34	0.95
C9—H9	0.95	C20—C21	1.512 (3)
C10—C11	1.391 (3)	C20—H20A	0.99
C10—H10	0.95	C20—H20B	0.99
C11—C12	1.389 (3)	C21—C22	1.395 (3)
C11—H11	0.95	C21—C26	1.397 (3)
C12—C13	1.387 (3)	C26—C25	1.387 (3)
C12—H12	0.95	C26—H26	0.95
C13—H13	0.95	C25—C24	1.390 (3)
C14—C19	1.396 (3)	C25—H25	0.95
C14—C15	1.402 (3)	C24—C23	1.385 (3)
C15—C16	1.381 (3)	C24—H24	0.95
C15—H15	0.95	C23—C22	1.387 (3)
C16—C17	1.393 (3)	C23—H23	0.95
C16—H16	0.95	C22—H22	0.95
			
P1—Pt1—Cl2	87.718 (19)	C18—C17—C16	119.4 (2)
P1—Pt1—Cl1	91.019 (19)	C18—C17—H17	120.3
Cl2—Pt1—Cl1	173.220 (18)	C16—C17—H17	120.3
P1—Pt1—P2	173.566 (18)	C17—C18—C19	120.6 (2)
Cl2—Pt1—P2	90.665 (19)	C17—C18—H18	119.7
Cl1—Pt1—P2	89.858 (19)	C19—C18—H18	119.7
C14—P1—C8	106.61 (9)	C18—C19—C14	120.17 (19)
C14—P1—C1	103.09 (9)	C18—C19—H19	119.9
C8—P1—C1	101.91 (9)	C14—C19—H19	119.9
C14—P1—Pt1	107.53 (7)	C32—C27—C28	118.98 (18)
C8—P1—Pt1	116.64 (7)	C32—C27—P2	123.67 (15)
C1—P1—Pt1	119.65 (7)	C28—C27—P2	117.34 (15)
C33—P2—C27	104.68 (9)	C31—C32—C27	120.15 (19)
C33—P2—C20	102.65 (9)	C31—C32—H32	119.9
C27—P2—C20	104.59 (9)	C27—C32—H32	119.9
C33—P2—Pt1	117.37 (7)	C32—C31—C30	120.3 (2)
C27—P2—Pt1	108.96 (7)	C32—C31—H31	119.8
C20—P2—Pt1	117.20 (7)	C30—C31—H31	119.8
C2—C1—P1	117.65 (14)	C29—C30—C31	119.70 (19)
C2—C1—H1A	107.9	C29—C30—H30	120.1
P1—C1—H1A	107.9	C31—C30—H30	120.1
C2—C1—H1B	107.9	C28—C29—C30	120.32 (19)
P1—C1—H1B	107.9	C28—C29—H29	119.8
H1A—C1—H1B	107.2	C30—C29—H29	119.8
C7—C2—C3	118.48 (19)	C29—C28—C27	120.50 (19)
C7—C2—C1	120.09 (18)	C29—C28—H28	119.7
C3—C2—C1	121.19 (18)	C27—C28—H28	119.7
C4—C3—C2	120.78 (19)	C34—C33—C38	119.19 (19)
C4—C3—H3	119.6	C34—C33—P2	119.13 (15)
C2—C3—H3	119.6	C38—C33—P2	121.60 (17)
C3—C4—C5	120.0 (2)	C33—C38—C37	119.6 (2)
C3—C4—H4	120	C33—C38—H38	120.2
C5—C4—H4	120	C37—C38—H38	120.2
C6—C5—C4	119.9 (2)	C36—C37—C38	120.6 (2)
C6—C5—H5	120.1	C36—C37—H37	119.7
C4—C5—H5	120.1	C38—C37—H37	119.7
C5—C6—C7	120.4 (2)	C35—C36—C37	120.0 (2)
C5—C6—H6	119.8	C35—C36—H36	120
C7—C6—H6	119.8	C37—C36—H36	120
C6—C7—C2	120.5 (2)	C36—C35—C34	119.8 (2)
C6—C7—H7	119.8	C36—C35—H35	120.1
C2—C7—H7	119.8	C34—C35—H35	120.1
C9—C8—C13	119.65 (19)	C33—C34—C35	120.8 (2)
C9—C8—P1	118.67 (15)	C33—C34—H34	119.6
C13—C8—P1	121.35 (16)	C35—C34—H34	119.6
C8—C9—C10	120.4 (2)	C21—C20—P2	115.37 (14)
C8—C9—H9	119.8	C21—C20—H20A	108.4
C10—C9—H9	119.8	P2—C20—H20A	108.4
C9—C10—C11	119.9 (2)	C21—C20—H20B	108.4
C9—C10—H10	120	P2—C20—H20B	108.4
C11—C10—H10	120	H20A—C20—H20B	107.5
C12—C11—C10	119.9 (2)	C22—C21—C26	118.42 (18)
C12—C11—H11	120	C22—C21—C20	120.11 (17)
C10—C11—H11	120	C26—C21—C20	121.43 (18)
C13—C12—C11	120.3 (2)	C25—C26—C21	120.50 (19)
C13—C12—H12	119.9	C25—C26—H26	119.8
C11—C12—H12	119.9	C21—C26—H26	119.8
C12—C13—C8	119.9 (2)	C26—C25—C24	120.50 (19)
C12—C13—H13	120.1	C26—C25—H25	119.8
C8—C13—H13	120.1	C24—C25—H25	119.8
C19—C14—C15	118.98 (19)	C23—C24—C25	119.4 (2)
C19—C14—P1	122.68 (15)	C23—C24—H24	120.3
C15—C14—P1	118.15 (15)	C25—C24—H24	120.3
C16—C15—C14	120.5 (2)	C24—C23—C22	120.2 (2)
C16—C15—H15	119.8	C24—C23—H23	119.9
C14—C15—H15	119.8	C22—C23—H23	119.9
C15—C16—C17	120.38 (19)	C23—C22—C21	120.96 (18)
C15—C16—H16	119.8	C23—C22—H22	119.5
C17—C16—H16	119.8	C21—C22—H22	119.5
			
Cl2—Pt1—P1—C14	83.74 (7)	C14—C15—C16—C17	1.8 (3)
Cl1—Pt1—P1—C14	−89.59 (7)	C15—C16—C17—C18	−0.8 (3)
Cl2—Pt1—P1—C8	−156.67 (7)	C16—C17—C18—C19	−0.1 (3)
Cl1—Pt1—P1—C8	30.00 (7)	C17—C18—C19—C14	0.0 (3)
Cl2—Pt1—P1—C1	−33.21 (8)	C15—C14—C19—C18	0.9 (3)
Cl1—Pt1—P1—C1	153.46 (8)	P1—C14—C19—C18	175.77 (17)
Cl2—Pt1—P2—C33	156.19 (7)	C33—P2—C27—C32	−101.79 (18)
Cl1—Pt1—P2—C33	−30.57 (7)	C20—P2—C27—C32	5.8 (2)
Cl2—Pt1—P2—C27	−85.16 (7)	Pt1—P2—C27—C32	131.87 (16)
Cl1—Pt1—P2—C27	88.08 (7)	C33—P2—C27—C28	78.23 (17)
Cl2—Pt1—P2—C20	33.27 (8)	C20—P2—C27—C28	−174.18 (16)
Cl1—Pt1—P2—C20	−153.49 (8)	Pt1—P2—C27—C28	−48.10 (17)
C14—P1—C1—C2	168.80 (15)	C28—C27—C32—C31	−0.5 (3)
C8—P1—C1—C2	58.37 (17)	P2—C27—C32—C31	179.56 (16)
Pt1—P1—C1—C2	−71.98 (16)	C27—C32—C31—C30	−0.4 (3)
P1—C1—C2—C7	100.76 (19)	C32—C31—C30—C29	0.3 (3)
P1—C1—C2—C3	−85.0 (2)	C31—C30—C29—C28	0.7 (3)
C7—C2—C3—C4	2.1 (3)	C30—C29—C28—C27	−1.6 (3)
C1—C2—C3—C4	−172.26 (18)	C32—C27—C28—C29	1.5 (3)
C2—C3—C4—C5	−0.3 (3)	P2—C27—C28—C29	−178.56 (16)
C3—C4—C5—C6	−1.4 (3)	C27—P2—C33—C34	−159.45 (16)
C4—C5—C6—C7	1.1 (3)	C20—P2—C33—C34	91.54 (17)
C5—C6—C7—C2	0.8 (3)	Pt1—P2—C33—C34	−38.53 (18)
C3—C2—C7—C6	−2.4 (3)	C27—P2—C33—C38	23.99 (19)
C1—C2—C7—C6	172.07 (18)	C20—P2—C33—C38	−85.02 (18)
C14—P1—C8—C9	157.60 (15)	Pt1—P2—C33—C38	144.91 (15)
C1—P1—C8—C9	−94.68 (16)	C34—C33—C38—C37	−1.2 (3)
Pt1—P1—C8—C9	37.51 (17)	P2—C33—C38—C37	175.32 (17)
C14—P1—C8—C13	−28.96 (18)	C33—C38—C37—C36	0.8 (3)
C1—P1—C8—C13	78.77 (17)	C38—C37—C36—C35	0.0 (4)
Pt1—P1—C8—C13	−149.04 (14)	C37—C36—C35—C34	−0.4 (3)
C13—C8—C9—C10	−0.8 (3)	C38—C33—C34—C35	0.9 (3)
P1—C8—C9—C10	172.78 (15)	P2—C33—C34—C35	−175.79 (16)
C8—C9—C10—C11	−0.7 (3)	C36—C35—C34—C33	−0.1 (3)
C9—C10—C11—C12	1.4 (3)	C33—P2—C20—C21	−63.36 (16)
C10—C11—C12—C13	−0.7 (3)	C27—P2—C20—C21	−172.43 (14)
C11—C12—C13—C8	−0.8 (3)	Pt1—P2—C20—C21	66.82 (16)
C9—C8—C13—C12	1.5 (3)	P2—C20—C21—C22	98.4 (2)
P1—C8—C13—C12	−171.87 (15)	P2—C20—C21—C26	−83.9 (2)
C8—P1—C14—C19	121.64 (18)	C22—C21—C26—C25	1.4 (3)
C1—P1—C14—C19	14.8 (2)	C20—C21—C26—C25	−176.4 (2)
Pt1—P1—C14—C19	−112.56 (17)	C21—C26—C25—C24	−0.3 (3)
C8—P1—C14—C15	−63.45 (18)	C26—C25—C24—C23	−0.5 (3)
C1—P1—C14—C15	−170.34 (16)	C25—C24—C23—C22	0.2 (3)
Pt1—P1—C14—C15	62.34 (17)	C24—C23—C22—C21	1.0 (3)
C19—C14—C15—C16	−1.8 (3)	C26—C21—C22—C23	−1.7 (3)
P1—C14—C15—C16	−176.92 (17)	C20—C21—C22—C23	176.08 (19)

### Hydrogen-bond geometry (Å, º)

*Cg*1, *Cg*2, *Cg*3 and *Cg*4 are the centroids of the C33—C38, C2—C7, C8—C13 and C27—C32 rings, repectively.

**Table:**

*D*—H···*A*	*D*—H	H···*A*	*D*···*A*	*D*—H···*A*
C7—H7···Cl2^i^	0.95	2.85	3.532 (2)	130
C26—H26···Cl2	0.95	2.72	3.555 (2)	147
C29—H29···Cl1^ii^	0.95	2.93	3.731 (2)	143
C16—H16···*Cg*1^ii^	0.95	2.73	3.443 (3)	132
C23—H23···*Cg*2^iii^	0.95	2.63	3.536 (2)	159
C29—H29···*Cg*3^ii^	0.95	2.99	3.525 (2)	117
C36—H36···*Cg*4^iv^	0.95	2.77	3.708 (3)	169

Symmetry codes: (i) −*x*+1, −*y*, −*z*+1; (ii) −*x*+1, −*y*+1, −*z*; (iii) −*x*, −*y*, −*z*+1; (iv) *x*−1, *y*, *z*.
